# Supplementary material for: Association between physical activity level and cardiovascular disease: An empirical analysis based on CHARLS data in 2018
Source: PLoS One. 2025 Aug 1;20(8):e0329232. doi: 10.1371/journal.pone.0329232 (PMC12316306; doi:10.1371/journal.pone.0329232)
Supplement: S1 File — (DOCX) [file pone.0329232.s001.docx]

| Characteristics | Total  (N=9015) | Non- CVD  (N=7946) | CVD  (N=1069) | *χ^2^* / *t* | *P* value |
| --- | --- | --- | --- | --- | --- |
| Age (years, M±SD) | 61.21±9.52 | 60.83±9.49 | 64.00±9.27 | -10.28 | <.001*** |
| Gender |  |  |  | 6.096 | .014* |
| Male | 4418（49.01） | 3932（49.48） | 486（45.46） |  |  |
| Female | 4597（50.99） | 4014（50.52） | 583（54.54） |  |  |
| Education |  |  |  | 2.229 | .328 |
| No formal  education | 1867（20.71） | 1628（20.49） | 239（22.36） |  |  |
| Primary education | 4163（46.18） | 3686（46.39） | 477（44.62） |  |  |
| Secondary education and above | 2985（33.11） | 2632（33.12） | 353（33.02） |  |  |
| Residence |  |  |  | 36.927 | <.001*** |
| City | 2392（26.53） | 2026（25.50） | 366（34.24） |  |  |
| Rural | 6623（73.47） | 5920（74.50） | 703（65.76） |  |  |
| Marital status |  |  |  | 4.912 | .027* |
| With spouse | 7755（86.02） | 6859（86.32） | 896（83.82） |  |  |
| Without spouse | 1260（13.98） | 1087（13.68） | 173（16.18） |  |  |
| Annual household consumption (M±SD) | 10.00±1.20 | 10.00±1.19 | 9.94±1.16 | 1.594 | .11 |
| Social Activities |  |  |  | 0.632 | .427 |
| With Social activities | 4797（53.21） | 4216（53.06） | 581（54.35） |  |  |
| No social activities | 4218（46.79） | 3730（46.94） | 488（45.65） |  |  |
| Depression |  |  |  | 70.647 | <.001*** |
| Suffer | 3598（39.91） | 3045（38.32） | 553（51.73） |  |  |
| Not suffering | 5417（60.09） | 4901（61.68） | 516（48.27） |  |  |
| Hypertension |  |  |  | 312.739 | <.001*** |
| Hypertension | 3104（34.43） | 2478（31.19） | 626（58.56） |  |  |
| Non-hypertension | 5911（65.57） | 5468（68.81） | 443（41.44） |  |  |
| Dyslipidemia |  |  |  | 320.448 | <.001*** |
| Dyslipidemia | 1667（18.49） | 1256（15.81） | 411（38.45） |  |  |
| Non-Dyslipidemia | 7348（81.51） | 6690（84.19） | 658（61.55） |  |  |
| Diabetes |  |  |  | 129.634 | <.001*** |
| Diabetes | 1010（11.20） | 780（9.82） | 230（21.52） |  |  |
| Non-Diabetes | 8005（88.80） | 7166（90.18） | 839（78.48） |  |  |
| Drinking status |  |  |  | 27.991 | <.001*** |
| Non-drinker | 3158（35.03） | 2861（36.01） | 297（27.78） |  |  |
| Current drinker | 5857（64.97） | 5085（63.99） | 772（72.22） |  |  |
| Smoking status |  |  |  | 16.453 | <.001*** |
| Non-smoker | 5145（57.07） | 4517（56.85） | 628（58.75） |  |  |
| Current smoker | 1007（11.17） | 858（10.80） | 149（13.94） |  |  |
| Former smoker | 2863（31.76） | 2571（32.36） | 292（27.32） |  |  |
| Non-smoker VS Current smoker  Non-smoker VS Former smoker  Current smoker VS Former smoker | | 0.024>0.05/3 | | 5.121 | 0.024 |
|  |  | 0.007<0.05/3 | | 7.286 | 0.007** |
|  |  | <.001*** | | 15.594 | <.001*** |
| PAL |  |  |  | 49.965 | <.001*** |
| Low PAL | 1236（13.71） | 1032（12.99） | 204（19.08） |  |  |
| Moderate PAL | 2195（24.35） | 1893（23.82） | 302（28.25） |  |  |
| High PAL | 5584（61.94） | 5021（63.19） | 563（52.67） |  |  |
| Low PAL vs Moderate PAL | | 0.029>0.05/3 | | 4.743 | 0.029 |
| Low PAL vs High PAL |  | <.001*** | | 41.820 | <.001*** |
| Moderate PAL vs High PAL | | <.001*** | | 21.545 | <.001*** |
|  |  |  |  |  |  |
| PAL quartile |  |  |  | 84.025 | <.001*** |
| Q1 <1732.5 | 2708（30.04） | 2286（28.77） | 422（39.48） |  |  |
| Q2 1732.5~4158 | 2080（23.07） | 1809（22.77） | 271（25.35） |  |  |
| Q3 4158~9198 | 2159（23.95） | 1934（24.34） | 225（21.05） |  |  |
| Q4 >9198 2068（22.94） | | 1917（24.13） 151（14.13） | |  |  |
| M±SD = mean ± standard deviation, **P*<.05, ***P*<.01, ****P*<.001. | | | | | |

|  |
| --- |
