# Supplementary material for: Association between physical activity level and cardiovascular disease: An empirical analysis based on CHARLS data in 2018
Source: PLoS One. 2025 Aug 1;20(8):e0329232. doi: 10.1371/journal.pone.0329232 (PMC12316306; doi:10.1371/journal.pone.0329232)
Supplement: Attachment — Submitted filename: Response to Reviewers-R1.docx [file pone.0329232.s003.docx]

Response to reviewers

We are so grateful for the thoughtful reviews provided by the external referees. We have tried to carefully address each recommendation. We believe that our manuscript is now stronger as a result of the modifications outlined below. We also thank the editoral staff and peer-reviewers for their contributions.

The modified text is highlighted in yellow in the revised manuscript. In addition to the specific responses to the referees shown below, we performed substantial language revising according reviews and editoral staff advise.

**Reviewer #1**

The referencing is not per the PLOS style and ref 23 authors name is somehow not correctly listed therefore would recommend to revisit the authors guideline (<https://journals.plos.org/plosone/s/submission-guidelines)> and proof read them as mentioned in the guideline ; In the text, cite the reference number in square brackets (We used the techniques developed by our colleagues [19] to analyze the data)

Response: We have carefully reviewed the PLOS ONE author guidelines and revised the references to ensure they fully comply with the journal’s style. Specifically, we have:

1. Corrected the formatting of all references to align with PLOS ONE requirements.
2. Verified and updated the author names in reference 23 to ensure accuracy.
3. Ensured that in-text citations are consistently formatted with square brackets, as per the guidelines (e.g., [19]).

Reference See line：410-611

**Reviewer #2**

General Comments

(1) - Methodological Limitations: Cross-sectional Design and Causality

The study employs a cross-sectional design, yet much of the discussion implies a causal relationship between PAL and CVD risk reduction. The wording throughout the manuscript should be tempered to avoid causal inferences. Statements such as; higher PAL reduces CVD risk; should be modified to indicate an association rather than causality. A clearer acknowledgment of reverse causality is necessary. Individuals with CVD may engage in less physical activity due to disease-related limitations, leading to an overestimation of the association. The discussion should explicitly recognize this issue and suggest prospective studies to confirm the findings.

Response:

- We have thoroughly reviewed the manuscript and modified all statements that may imply causality. For example, "higher PAL reduces the risk of adverse outcomes" has been revised to "higher PAL is associated with a lower risk of adverse outcomes." See line：299-302
- In response to your suggestions, a discussion on the reverse relationship between physical activity and cardiovascular disease has been added to the discussion section. It is also recommended that future studies adopt a prospective design to better elucidate the association between PAL and CVD risk. For discussion additions, See line：316-320

(2)- Confounding Factors and Model Adjustments

The multivariate analysis lacks adjustment for key confounders, including hypertension, diabetes, hyperlipidemia, smoking, alcohol consumption, and BMI; all of which are strong predictors of CVD and could mediate the association with PAL.

Without adjusting for these critical variables, it remains unclear whether the observed association is due to PAL itself or reflects a clustering of other healthier lifestyle behaviors in the high PAL group. The authors should include additional confounders in the regression models or justify why these variables were excluded. The impact of social and economic status (which may influence both PAL and access to healthcare) should be discussed more explicitly as a potential source of residual confounding.

Response:

- In this study, we re-analyzed the multivariate regression model by incorporating the covariates hypertension, dyslipidemia, diabetes, drinking status, smoking status. These variables were available in CHARLS dataset, and their inclusion provided a more comprehensive perspective for assessing the relationship between PAL and CVD risk. Additionally, we considered the inclusion of potential confounding factors such as BMI, high-density lipoprotein cholesterol (HDL-C), low-density lipoprotein cholesterol (LDL-C), and triglycerides (TG) to further control for confounding effects. However, due to the lack of physical examination and blood test data in the 2018 CHARLS dataset, these variables were ultimately not included in the analysis. In the limitations, we recommend that future studies incorporate these variables to enable a more comprehensive evaluation of the relationship between PAL and CVD risk. For further details on the CHARLS database, please refer to its official website : <https://charls.pku.edu.cn/>. For newly added variables, See line : 221. For limitations additions, See line：356-361.
- Furthermore, although this study did not include socioeconomic status (SES) as a separate covariate in the analysis, we incorporated both educational attainment and household consumption expenditure, which are well-established indicators of SES and reflect different dimensions of socioeconomic status. We have also acknowledged the potential confounding effect of SES in the limitations section of the manuscript. Finally, in accordance with your suggestion, we have explicitly added a discussion in the discussion section regarding the impact of social and economic status on PAL and access to healthcare. For discussion additions, See line：320-331. For limitations additions, See line：360.

References on Education and Consumption Level as Indicators of Socioeconomic Status:

- 51.	Broer M, Bai Y, Fonseca F. A Review of the Literature on Socioeconomic Status and Educational Achievement. In: Socioeconomic Inequality and Educational Outcomes. Springer; 2019:7-17. doi:10.1007/978-3-030-11991-1_2.
- 52.	Poirier MJP. Systematic comparison of household income, consumption, and assets to measure health inequalities in low- and middle-income countries. Sci Rep. 2024;14(1):3851. E pub 20240215. Doi : 10.1038/s41598-024-54170-1. PubMed PMID: 38360925; PubMed Central PMCID: PMCPMC10869835.

(3)- Self-Reported CVD Diagnosis: Accuracy and Bias

The diagnosis of CVD is based on self-reported physician-diagnosed heart disease or stroke. This method is prone to recall bias and may lead to misclassification errors. Participants with undiagnosed CVD or those with subjective symptoms but no formal diagnosis may be misclassified. The sensitivity and specificity of self-reported CVD data should be addressed in the discussion, and comparisons with objectively measured CVD prevalence in similar populations should be provided if available.

Response:

- In accordance with your suggestion, this study explicitly acknowledges the limitations associated with self-reported diagnoses of CVD. See line：361-364. However, Xie (2019). demonstrated that 77.5% of self-reported coronary heart disease cases were consistent with medical records in the English Longitudinal Study of Ageing (ELSA). Similarly Glymour (2009). found that misreporting of stroke was non-systematic, and self-reported stroke data could be reliably used to study the incidence and risk factors of stroke in the Health and Retirement Study (HRS). These findings suggest that the potential misclassification bias is likely minimal. Furthermore, self-reported CVD diagnoses have been widely used in various scientific studies.

Related references:

- Xie W, Zheng F, Yan L., Zhong B. Cognitive decline before and after incident coronary events. J Am Coll Cardiol. 2019;24:3041–3050. Doi: 10.1016/j.jacc.2019.04.019.
- Glymour M M, Avendano M. Can Self-Reported Strokes Be Used to Study Stroke Incidence and Risk Factors? [J/OL]. Stroke, 2009, 40(3): 873-879. DOI:10.1161/STROKEAHA.108.529479.
- He D, Wang Z, Li J, Yu K, He Y, He X, et al. Changes in frailty and incident cardiovascular disease in three prospective cohorts. Eur Heart J. 2024;45(12):1058-68. Doi : 10.1093/eurheartj/ehad885. PubMed PMID: 38241094.
- Yang Y, Cao L, Xia Y, Li J. The effect of living environmental factors on cardiovascular diseases in Chinese adults: results from a cross-sectional and longitudinal study. Eur J Prev Cardiol. 2023;30(11):1063-73. Doi : 10.1093/eurjpc/zwac304. PubMed PMID: 3653.
- The prevalence of CVD has been compared in the Discussion section. Our study found an 11.86% prevalence of CVD among middle-aged and elderly populations, which is similar to the findings of Yuxiang W (2023). For discussion additions, See line：257-266.
- Yuxiang W, Rulin M, Heng G, Xianghui Z, Jia H, Xinping W, et al. Prevalence of cardiovascular disease and its influencing factors among the Uygur population in rural areas of Xinjiang. Chinese Journal Of Disease Control & Prevention. 2023;27(4):385-91. Doi : 10.16462/j.cnki.zhjbkz.2023.04.003.

(4) PAL Categorization and MET-Minute Thresholds

The classification of low, moderate, and high PAL is based on arbitrary MET-minute cutoffs (<600, 600-3000, >3000 MET-min/week). However, it is unclear whether these categories align with established guidelines such as WHO, ACSM, or AHA recommendations. Justification should be provided for the chosen MET cutoffs. Alternatively, the analysis could be repeated using quartiles or deciles of PAL to explore whether different thresholds yield different results. The interpretation of 3000 MET-min/week as a threshold for reduced CVD prevalence should be critically examined, as it does not align directly with WHO recommendations for physical activity.

Response:

- MET-minute/week cutoffs

The MET-minutes/week cutoffs (<600, 600–3000, and >3000 MET-min/week) were selected based on the classification used in the International Physical Activity Questionnaire (IPAQ). This method of physical activity categorization has been widely adopted in various studies. Below are references to studies that have utilized this classification.

In studies conducted by Lear (2017), Bauman (2009), and Dąbrowska (2019) et al., physical activity levels were assessed using the International Physical Activity Questionnaire (IPAQ) and categorized into low, moderate, and high, consistent with the classification method used in this study. Lear (2017) found that moderate and high physical activity levels were associated with a graded reduction in cardiovascular disease (CVD) mortality compared to low levels, while Dąbrowska et al. reported that women with high and moderate activity levels experienced milder menopausal symptoms compared to inactive counterparts.

Related references:

- Lear SA, Hu W, Rangarajan S, et al. The effect of physical activity on mortality and cardiovascular disease in 130 000 people from 17 high-income, middle-income, and low-income countries: the PURE study published correction appears in Lancet. 2017 Dec 16;390(10113):2626. doi: 10.1016/S0140-6736(17)32596-5.
- Bauman A, Bull F, Chey T, et al. The International Prevalence Study on Physical Activity: results from 20 countries. Int J Behav Nutr Phys Act. 2009;6:21. Published 2009 Mar 31. doi:10.1186/1479-5868-6-21.
- Dąbrowska-Galas M, Dąbrowska J, Ptaszkowski K, Plinta R. High Physical Activity Level May Reduce Menopausal Symptoms. Medicina (Kaunas). 2019;55(8):466. Published 2019 Aug 11. doi:10.3390/medicina55080466.
- PAL quartile

We conducted supplementary multiple logistic regression analyses using quartiles of PAL, and the results showed trends consistent with those observed in the three-category classification of physical activity. See line: 217.

Baseline characteristics of physical activity quartile and comparisons of differences between groups can be found in Supplementary File S1.

- To 3000 MET-min/week as a threshold

In Model 2, after adjusting for hypertension, dyslipidemia, diabetes, drinking status, smoking status, both the moderate PAL (600-3000 MET minutes/week) and high PAL groups exhibited lower risk levels, whereas in the previous model, only the high PAL group (>3000 MET minutes/week) showed a lower risk. These findings align with the results reported by Lear et al. (2017).

Lear et al(2017). physical activity levels were assessed using the International Physical Activity Questionnaire (IPAQ) across 14 countries with varying economic statuses. Total physical activity was categorized into low (<600 MET-minutes/week), moderate (600–3000 MET-minutes/week), and high (>3000 MET-minutes/week). The study found that moderate and high physical activity levels were associated with a graded reduction in cardiovascular disease (CVD) mortality compared to low physical activity levels (<600 MET-minutes/week).

- Lear SA, Hu W, Rangarajan S, et al. The effect of physical activity on mortality and cardiovascular disease in 130 000 people from 17 high-income, middle-income, and low-income countries: the PURE study published correction appears in Lancet. 2017 Dec 16;390(10113):2626. Doi: 10.1016/S0140-6736(17)32596-5.

The findings of our study partially align with the WHO and ACSM recommended physical activity levels of 600-1200 MET-minutes/week. The discrepancy may arise because the physical activity levels recommended by WHO and ACSM refer to the amount of activity required in addition to daily lifestyle activities, whereas the physical activity levels in this study include non-exercise daily physical activities. Furthermore, most current studies suggest that exceeding the physical activity levels recommended by the WHO and ACSM guidelines is more beneficial than merely meeting these recommendations.

- Williams PT. Dose-response relationship of physical activity to premature and total all-cause and cardiovascular disease mortality in walkers. PLoS One. 2013;8(11):e78777. Published 2013 Nov 29. doi:10.1371/journal.pone.0078777
- Thompson D, Batterham AM, Peacock OJ, Western MJ, Booso R. Feedback from physical activity monitors is not compatible with current recommendations: A recalibration study. Prev Med. 2016; 91: 389-394.doi: 10.1016/j. ypmed. 2016.06.017.

(5)- Restricted Cubic Spline Interpretation

The restricted cubic spline analysis suggests a linear inverse association between PAL and CVD (p>0.05 for non-linearity). However, Figure 2 should be more clearly annotated to indicate where the confidence intervals widen, which could suggest statistical uncertainty at extreme PAL levels. The authors should discuss whether this linear trend holds across all PAL levels or whether the association plateaus at higher MET levels, as some studies suggest diminishing returns beyond a certain point.

Response:

- Figure 2 caption

In the Figure 2, the OR values are represented by the solid red line, with the 95% confidence intervals (95% CI) indicated by the shaded gray area. the overall trend of the curve demonstrates a negative correlation, with OR values decreasing as amount of physical activity increase. This indicates that higher levels of physical activity are associated with a lower risk of CVD prevalence. At weekly physical activity levels exceeding10,000 MET-minutes/week, the curve begins to plateau, suggesting that the additional health benefits of further increases in physical activity may diminish. Furthermore, the confidence intervals were narrow at moderate and high physical activity levels, indicating greater statistical stability. However, at extreme MET-minutes/week values (<600 or >12,000), the confidence intervals wide, reflecting increased uncertainty due to fewer data points within these ranges. For figure note additions, See line：227-235、238-243.

- linear trend holds across all PAL levels

The restricted cubic spline plot revealed a significant inverse linear trend between weekly physical activity levels (ranging from 0 to 15,000 MET-minutes/week) and CVD risk. Specifically, higher levels of physical activity are consistently associated with a lower risk of CVD prevalence. The odds ratio demonstrated a linear decline as physical activity levels increased. However, the curve began to plateau when physical activity exceeded 10,000 MET-minutes/week. This phenomenon suggests the potential existence of a threshold effect in the relationship between physical activity and CVD risk. Specifically, further increases in physical activity beyond a certain level may not significantly contribute to additional reductions in CVD risk. This finding aligns with existing research, which indicates that the health benefits of physical activity may plateau beyond a certain threshold, and excessive exercise could potentially introduce other health risks. For discussion additions, See line：282-291.

(6)- Generalizability and Population-Specific Considerations

The study is based on Chinese middle-aged and elderly individuals, and lifestyle patterns may differ significantly from Western populations. The discussion should explicitly address the external validity of the findings and whether they can be generalized to younger individuals, different ethnic groups, or non-Asian populations.

Urban-rural differences in occupational physical activity should also be discussed, as many rural participants may engage in high PAL due to labor-intensive work rather than structured exercise.

Response:

- External validity

The findings of this study are based on a cohort of middle-aged and elderly individuals in China, and it is important to consider the extent to which these results can be generalized to other populations. However, notable differences exist in physical activity patterns between China and Western countries. In Western countries, particularly in Europe and North America, structured forms of exercise-such as gym-based activities, aerobic classes, and organized fitness programs-are more prevalent. These activities are typically planned, organized, and primarily aimed at improving health and physical fitness. As highlighted by Hallal(2012), physical activity in Western populations is more closely associated with structured exercise. In contrast, in China, physical activity is predominantly linked to daily living activities and occupational labor rather than structured exercise. These differences may influence the observed associations between PAL and CVD risk. Although the dose-response relationship observed in this study aligns with findings from Western populations, caution should be exercised when generalizing these results to younger individuals, different ethnic groups, or non-Asian populations. For discussion additions, See line：332-344.

- Hallal PC, Andersen LB, Bull FC, Guthold R, Haskell W, Ekelund U. Global physical activity levels: surveillance progress, pitfalls, and prospects. Lancet. 2012;380(9838):247-57. Doi : 10.1016/s0140-6736(12)60646-1. PubMed PMID: 22818937.
- Lear SA, Hu W, Rangarajan S, Gasevic D, Leong D, Iqbal R, et al. The effect of physical activity on mortality and cardiovascular disease in 130 000 people from 17 high-income, middle-income, and low-income countries: the PURE study. Lancet. 2017;390(10113): 2643-54. E pub 20170921. Doi : 10.1016/s0140-6736(17)31634-3. PubMed PMID: 28943267.
- Urban-rural differences in occupational physical activity

Li J (2024), has demonstrated significant differences in occupational physical activity (OPA) patterns between urban and rural areas in China. In rural areas, participants are more likely to engage in labor-intensive work, such as agricultural labor, which requires substantial physical exertion. In contrast, urban participants are more often employed in office-based or other low-physical-activity occupations. Rural adults exhibit higher levels of occupational moderate-to-vigorous physical activity (MVPA), which is significantly associated with lower risks of all-cause and CVD mortality. Conversely, urban areas show lower levels of occupational MVPA, and no significant association with mortality is observed. These findings underscore the importance of urban-rural differences in the relationship between OPA and health outcomes. For discussion additions, See line：344-353.

- Li J, Zhang X, Zhang M, Wang L, Yin P, Li C, et al. Urban-rural differences in the association between occupational physical activity and mortality in Chinese working population: evidence from a nationwide cohort study. Lancet Reg Health West Pac. 2024;46:101083. Epub 20240507. Doi : 10.1016/j.lanwpc.2024.101083. PubMed PMID: 38745972; PubMed Central PMCID: PMCPMC11091516.

Detailed Comments

(1) Abstract

The phrase: high PAL reduces CVD risk; should be changed to; high PAL is associated with a lower CVD prevalence. The sample size (9015 participants) should be explicitly stated in the methods section of the abstract.

Response:

- We have thoroughly reviewed the manuscript and modified all statements that may imply causality. For example, "higher PAL reduces the risk of adverse outcomes" has been revised to "higher PAL is associated with a lower risk of adverse outcomes." See line：299-302.
- Additionally, the sample size of 9015 participants has now been explicitly stated in the abstract. See line：34.

(2) Introduction

The introduction effectively highlights the importance of PAL, but it oversimplifies the causal relationship. Statements such as "maintaining >3000 MET-min/week reduces CVD risk" should be softened to "is associated with lower CVD prevalence."

Response: We have thoroughly reviewed the introduction section of the manuscript and revised all statements that might imply causality. For example, the statement" when physical activity exceeds 7258 MET-min/week, the likelihood of adverse outcomes in CVD patients is significantly reduced "has been modified to" when physical activity exceeds 7,258 MET-min/week, it is associated with a lower risk of adverse outcomes in patients with cardiovascular disease." See line：89-91.

(3) Methods

Multivariable model adjustments: Include key covariates such as hypertension, diabetes, smoking, alcohol consumption, and BMI.

Clarify PAL measurement: Was PAL derived solely from self-reported surveys, or were objective measurements (e.g., accelerometers) available?

Describe how missing data were handled: Was multiple imputation used, or were missing cases excluded?

Response：

- In this study, we re-analyzed the multivariate regression model by incorporating covariates such as hypertension, diabetes, hyperlipidemia, smoking status, and alcohol consumption. See line：217.
- In this study, the primary exposure variable was physical activity, assessed through a self-reported questionnaire. See line：114-115.
- Based on existing literature, cases with missing data that met the exclusion criteria were excluded. See line：107-111.
- Liang XZ, Chai JL, Li GZ, et al. A fall risk prediction model based on the CHARLS database for older individuals in China. BMC Geriatr. 2025;25(1):170. Published 2025 Mar 13. doi:10.1186/s12877-025-05814-y

(4) Results：

Table 1: Add p-values for baseline characteristics to indicate whether significant differences exist between groups.

Table 3: Clarify whether PAL was modeled as a categorical or continuous variable in the logistic regression.

Response: The P-values for baseline characteristics have been added to S1 Supplementary file, and it has been clarified in Table 3 that PAL was treated as a categorical variable in the logistic regression analysis. See line：218-219.

(5) Discussion：

Causality: Strengthen the acknowledgment of reverse causality and residual confounding.

Confounding bias: Explicitly discuss the lack of adjustment for key clinical variables such as hypertension, smoking, and diabetes.

Dose-response relationship: Discuss whether there is a plateau effect for high PAL levels.

Generalizability: Address whether findings are applicable to younger or non -Chinese populations.

Response: We have addressed the reviewer's concerns by strengthening the acknowledgment of potential reverse causality and residual confounding, explicitly discussing the lack of adjustment for key clinical variables such as hypertension, smoking, and diabetes, exploring the possibility of a plateau effect at high PAL levels in the dose-response relationship, and clarifying the generalizability of our findings to younger or non - Chinese populations. These revisions have been incorporated into the revised manuscript to enhance the clarity and robustness of our analysis. See line：316-320、158-164、282-291、332-344.

(6) Figures and Tables

Figure 2: Add confidence interval shading to highlight the uncertainty at extreme PAL levels.

Table 3: Consider including effect sizes (ORs) for additional quartiles or deciles of PAL instead of only three broad categories.

Response: We have addressed as follows: (1) Confidence interval shading has been added to Figure 2 to highlight the uncertainty at extreme PAL levels; (2) Effect sizes (ORs) for additional quartiles of PAL have been included in Table 3 to provide a more detailed analysis beyond the three broad categories. These revisions have been incorporated to enhance the clarity and precision of the results. See line：241-242、217.

We hope these revisions address the reviewer’s concerns and improve the overall quality of the manuscript. Thank you again for your valuable feedback.

Sincerely,

HAIWEI LI
