# Supplementary material for: Association between physical activity level and cardiovascular disease: An empirical analysis based on CHARLS data in 2018
Source: PLoS One. 2025 Aug 1;20(8):e0329232. doi: 10.1371/journal.pone.0329232 (PMC12316306; doi:10.1371/journal.pone.0329232)
Supplement: Attachment — Submitted filename: Response to reviewers 2.docx [file pone.0329232.s004.docx]

Response to reviewers

We are so grateful for the thoughtful reviews provided by the external referees. We have tried to carefully address each recommendation. We believe that our manuscript is now stronger as a result of the modifications outlined below. We also thank the editoral staff and peer-reviewers for their contributions.

Revisions in the manuscript are highlighted in yellow. In addition to the specific responses to reviewers below, we have made revisions to grammar and tense based on suggestions from the reviewers and editors.

**Reviewer #2**

Weaknesses (Major issues)

(1) -Handling of residual confounding (especially socioeconomic status) is insufficiently robust.

Response：We sincerely appreciate the reviewer's insightful comments regarding potential residual confounding related to socioeconomic status (SES). In line with current methodological standards in the literature, we have reconceptualized the assessment of socioeconomic status (SES) through：

Socioeconomic status was comprehensively assessed through two key dimensions: educational attainment and per capita household expenditure. First, participants' highest educational level was quantified using standardized values (illiterate/semiliterate = 0, sishu/elementary school = 6, middle school = 9, high school/vocational school = 12, college = 16, postgraduate = 19). Second, per capita expenditure was calculated by dividing total household expenditure by the number of household members. To account for scale differences, both education level and per capita expenditure were standardized using Z-scores, and these standardized values were summed to create a composite SES score, with higher values indicating higher SES status. Participants were then stratified into three groups using tertile categorization: low SES (bottom tertile), medium SES (middle tertile), and high SES (top tertile), ensuring balanced group sizes. See line：159-169.

The robustness of our primary findings persisted following SES covariate adjustment (PAL：[Model 2: Moderate PALs: OR=0.79,95% CI: 0.64-0.97, P<0.05, High PALs: OR=0.72, 95% CI: 0.60~0.87, P<0.05]；PAL quartile：[Model 2：Q2:1733~4158,OR=0.90,95% CI：0.75-1.07，P=0.225，Q3:4158~9198,OR=0.78,95% CI：0.65-0.94，P<0.05，Q4:>9198,OR=0.64,95% CI：0.52-0.79，P<0.001]) Moreover, in accordance with your recommendations, we have explicitly acknowledged in the Limitations section the potential for residual confounding arising from unmeasured factors including, but not limited to: diet, medication adherence, genetic factors. See line：216-218、395.

Socioeconomic Status References:

- Li W, Zhang X, Gao H, Tang Q. Heterogeneous effects of socio-economic status on social engagement level among Chinese older adults: evidence from CHARLS 2020. Front Public Health. 2024;12:1479359. Published 2024 Nov 29. doi:10.3389/fpubh.2024.1479359

(2) -The definition of PAL categories (low, moderate, high) remains somewhat arbitrary despite partial justification. Some new additions (e.g., quartile analyses) are insufficiently integrated into the discussion.

Response：We sincerely appreciate your valuable feedback on the research methodology and integration of results. We fully agree with your perspective that the classification of physical activity levels (PAL) requires more thorough justification and have made the following improvements to address the insufficient incorporation of the new quartile analysis in the discussion section.

Currently, there are multiple classification methods for physical activity levels. In this study, we adopted a grouping approach based on existing literature, which may still appear somewhat arbitrary. Notably, following your suggestion, we conducted supplementary quartile analyses, which revealed a more nuanced dose-response relationship between physical activity levels (PAL) and CVD risk. As physical activity increased from Q1 (<1,733 MET-minutes/week) to Q4 (>9,198 MET-minutes/week), the odds ratios (ORs) for CVD risk demonstrated a clear graded decline (OR decreasing from 0.90 to 0.64, P for trend <0.05). This monotonic decreasing trend further supports the protective effect of physical activity. Specifically, compared to the least active group (Q1), the Q2 group (1,733–4,158 MET-minutes/week) showed a 10% reduction in risk (OR = 0.90, 95% CI: 0.75–1.07), the Q3 group (4,158–9,198 MET-minutes/week) exhibited a significant 22% decrease (OR = 0.78, 95% CI: 0.65–0.94), and the Q4 group demonstrated the greatest reduction at 36% (OR = 0.64, 95% CI: 0.52–0.79). Although the three-category and quartile analyses used different cutoff points, both approaches consistently demonstrated a similar decreasing trend." This concordance across models strengthens the credibility of our findings. These results provide valuable insights for developing more precise exercise recommendations, suggesting that exceeding minimum activity guidelines may yield additional health benefits. However, optimal activity thresholds may vary across populations. See line：282-292.

Additionally, newly incorporated covariates—including hypertension, diabetes, dyslipidemia, smoking, alcohol consumption, and socioeconomic status—were integrated into the discussion, as these factors are also strongly associated with CVD risk. See line：274-279.

(3) -Plateau Effect：The narrative on plateau effects in the dose-response relationship needs more depth. You mention a plateau at >10,000 MET-min/week, but the biological plausibility and comparison to other cohort studies (e.g., PURE, UK Biobank) should be discussed. Also, briefly speculate on whether very high levels of activity might confer risks (overtraining, cardiovascular stress).

Response：From a biological mechanism perspective, the health benefits of physical activity typically follow a nonlinear dose-response curve. Regular, moderate exercise can significantly reduce the risk of various chronic diseases by improving insulin sensitivity, reducing systemic inflammation, and enhancing cardiopulmonary function. However, when physical activity exceeds a certain threshold, the body's metabolic and organ systems reach relative saturation, potentially leading to diminishing health returns. This manifests as a plateau effect where the magnitude of risk reduction gradually stabilizes. See line：310-316.

A large cohort study from PURE (Prospective Urban Rural Epidemiology) demonstrated graded reductions in mortality (HR 0.80, 95% CI 0.74-0.87 and 0.65, 0.60-0.71; p-trend<0.0001) and major CVD (0.86, 0.78-0.93; p-trend<0.001) when comparing moderate (600-3000 MET-minutes/week) and high (>3000 MET-minutes/week) activity levels against low activity (<600 MET-minutes). Another UK Biobank study identified significant linear dose-response relationships between moderate/vigorous/total physical activity levels and CVD risk. Additionally, the study by Bakker EA et al, identified a dose-response relationship between PAL, major adverse cardiovascular events (MACE), and all-cause mortality across different population groups: healthy individuals, individuals with cardiovascular risk factors(CVRF),and individuals with CVD. PAL, measured in MET-minutes per week, was divided into quartiles: Q1 (1–1912 MET-minutes/week), Q2 (1913–3690 MET-minutes/week), Q3 (3691–7257 MET-minutes/week), and Q4 (>7258 MET-minutes/week). The study found that higher PAL was associated with a lower risk of adverse outcomes in both healthy individuals and patients with CVRF. For CVD patients, only the highest PA quartile was associated with a lower risk of adverse prognosis. See line：319-333.

However, extremely high activity levels may not be absolutely safe. Research indicates that sustained excessive exercise may trigger overtraining syndrome, potentially inducing learning and memory impairments through elevated inflammatory cytokines and oxidative stress markers. Additionally, studies report that chronic endurance athletes may develop atrial dilation and increased arrhythmia risk. In summary, physical activity exceeding 10,000 MET-minutes/week may reach a "physiological saturation point" - while not immediately hazardous, the health benefits plateau. Beyond this threshold, certain individuals may experience diminishing returns or potential adverse effects with further training load escalation. See line：339-346.

(4) -Figure 2 still lacks enough annotation to guide readers in interpreting wide confidence intervals.

Response：As per your suggestion, a detailed figure legend has been added below Figure 2 to help readers understand the wide confidence intervals.

Figure caption: Association between amount of physical activity and cardiovascular disease risk. This figure presents the results of RCS analysis based on Model 2, illustrating the dose-response relationship between physical activity (measured in MET-minutes/week) and the OR for cardiovascular disease. The OR values are represented by the solid red line, with the 95% confidence intervals (95% CI) indicated by the shaded gray area. The graph is truncated at 15,000 MET-minutes/week, corresponding to the 93rd percentile of the physical activity distribution. The red vertical dashed lines indicated key thresholds at 600, 3,000, 10,000, and 12,000 MET-minutes per week. The confidence intervals were wider at low (<600 MET-minutes/week) and high (>12,000 MET-minutes/week) levels of physical activity, which may be due to smaller sample sizes or greater data variability at these extremes. In contrast, within the 3,000–10,000 MET-minutes/week range, the sample size was larger and more stable, resulting in narrower confidence intervals. Overall, the trend showed that the risk ratio decreased progressively with increasing physical activity. See line：245-251.

Detailed Comments

Introduction

Good improvement on causal language. However, some remaining sentences still sound too causal (e.g., “maintaining moderate-to-high physical activity reduces the burden of CVD”). Please revise consistently to “associated with”.

Response：Thank you for your careful review and for pointing this out. We are grateful for your acknowledgment of the improvements made and fully agree on the importance of avoiding causal language in observational studies. Accordingly, we have thoroughly examined the manuscript's introduction section and revised all remaining instances that might contain causal implications. For example, the original statement: 'Research shows that, compared to participants reporting no leisure-time physical activity, those who engaged in guideline-recommended minimum levels of physical activity—500 MET-minutes/week according to the 2008 US guidelines—experienced modest lower CVD risk' has been modified to: 'Research shows that, compared to participants reporting no leisure-time physical activity, those meeting the minimum physical activity level recommended by the 2008 U.S. guidelines (500 MET-minutes/week) were associated with a lower CVD risk.' All similar expressions throughout the manuscript have been corrected to reflect associations rather than causal relationships." See line：76-82.

Methods

Exposure Measurement:

The reliance solely on self-reported PAL should be more critically discussed upfront. Objective measures (e.g., accelerometer) are unavailable, but the authors could mention this as a limitation in the Methods itself (not only in Discussion).

Response：According to your suggestion, this limitation has been added in the methodology section: PAL is assessed through self-report questionnaires. However, Objective tools such as accelerometer were not employed, which may introduce potential bias due to inaccurate recall or reporting. See line：111-112.

Missing Data Handling:

The handling of missing data was by case-wise deletion. Given the substantial exclusions (over 10,000 participants), please add a sensitivity analysis or at least discuss the potential for selection bias more explicitly in the Discussion section.

Response：To verify the robustness of our primary findings, we conducted sensitivity analyses by reclassifying physical activity levels (PAL) from the original three-category grouping (low/medium/high) into quartiles (Q1-Q4), as suggested by your advice. The results demonstrated consistent trends between the quartile-based and three-category analyses (Table 3), supporting the reliability of our main findings. We have incorporated the discussion of these quartile-based analyses in the manuscript. See line：282-292. Additionally, we have included a discussion of potential selection bias in the limitations section to more explicitly address the possibility of such bias. See line：399-402.

Covariate Selection:

While education and household consumption were used as proxies for socioeconomic status (SES), the absence of an explicit SES composite variable is a concern. Discuss this more clearly. Alternatively, re-analyze with household consumption quintiles or categories if possible.

Response：Following your recommendation and consistent with established methodological standards in the literature, we redefined socioeconomic status (SES) and included it as a covariate in the logistic regression analyses. The robustness of our primary findings remained after SES adjustment, with full results detailed in Response to Comment 1. See line：159-169.

Results

Figure 2 (Restricted Cubic Spline):

While the figure is technically correct, the wide confidence intervals at high PAL (>12,000 MET-min/week) and low PAL (<600 MET-min/week) need explicit labels or annotations. Suggest adding dotted vertical lines at 600, 3000, and 10,000 MET-minutes/week to show thresholds. Briefly describe in the Results section when the OR curve begins to plateau (currently mixed into the Discussion only).

Response：As requested, we have added dashed lines at 600, 3,000, 10,000, and 12,000 MET-minutes/week in Figure 2 to indicate threshold values, along with clear labels and annotations in the figure legend. Additionally, we have included a description in the Results section specifying when the OR curve begins to plateau. See line：245-251、231-233.

Discussion

Residual Confounding:

The potential for residual confounding (especially unmeasured variables like diet, medication adherence, genetic factors) should be acknowledged.

Response：Per your suggestion, we have added diet, medication adherence, and genetic factors as potential residual confounders in the Limitations section. See line：395.

Current references suggesting minimal misclassification for self-reported CVD are appreciated but still optimistic; please explicitly note that this might not fully apply to the Chinese CHARLS cohort.

Response：As per your suggestion, we have added the following content to the manuscript: Although prior studies indicate a relatively low misclassification rate for self-reported CVD, these findings should be interpreted with caution given the substantial proportion of rural participants in the CHARLS cohort in China, along with their limited CVD awareness and potential recall bias, which may increase misclassification risk. See line：413-416.

Plateau Effect:

You mention a plateau at >10,000 MET-min/week, but the biological plausibility and comparison to other cohort studies (e.g., PURE, UK Biobank) should be discussed.

Also, briefly speculate on whether very high levels of activity might confer risks (overtraining, cardiovascular stress).

Response：Per your suggestion, we have added the following content to the Discussion section: (1) the biological mechanisms underlying our observational findings, (2) comparisons with results from other cohort studies, and (3) preliminary speculation about potential adverse effects associated with extremely high physical activity levels. Please see Response 3 for details. See line：310-316、319-333、339-346.

Generalizability:

Good discussion on urban/rural differences and cultural factors. It would be helpful to strengthen the point that findings might not apply to populations where leisure-time physical activity (rather than occupational activity) predominates.

Response：We sincerely appreciate the reviewer's positive feedback regarding our discussion of urban-rural disparities and cultural factors. We fully concur with the insightful observation that our findings may not be entirely generalizable to populations where leisure-time physical activity predominates. As suggested, we have supplemented the Discussion section as follows In our cohort, rural residents accounted for 73.47% of participants compared to 26.53% urban dwellers. Consequently, the applicability of our findings may be limited in populations where leisure-time physical activity serves as the primary form of exercise. See line：385-388.

Minor Points

Proofreading is still needed:

Fix minor English grammar issues (e.g., "the confidence intervals wide" → "the confidence intervals widened"). Consistent tenses (prefer "was/were" rather than "is/are" when describing past data).

Response：We thank the reviewer for their careful reading. We have thoroughly reviewed the manuscript and corrected all identified grammatical issues, including verb tense consistency (using past tense for results) and syntax errors (e.g., "confidence intervals wide" → "widened"). All changes have been tracked in the revised manuscript. See line：236.
